# Can datasets from long-term biomonitoring programs detect climate change effects on stream benthos?

**DOI:** 10.1177/00368504231219335

**Published:** 2023-12-17

**Authors:** Robert C. Bailey, Trefor B. Reynoldson

**Affiliations:** 85458 Ontario Tech University, Faculty of Science, Oshawa, Ontario, Canada

**Keywords:** Climate change, reference condition approach, bioassessment, biomonitoring, stream ecosystems, benthic invertebrates, temporal variability

## Abstract

We analyzed datasets from a long-term monitoring program of stream ecosystems in British Columbia, Canada, to determine whether or not it could detect climate change effects. In the Fraser River Basin (monitoring timespan 1994-2019), there was a marked (∼50%) increase in alpha diversity in reference streams, while BC North Coast (2004–2021) streams showed a modest trend of decreasing diversity and Columbia River Basin (2003–2018) and Vancouver Island (2001–2019) streams showed modestly increasing diversity. In all four regions, diversity across all sites in a specific period was primarily a function of sampling effort during this period rather than a temporal trend. Across all the regions, only three of 21 groups of faunally similar sites defined by Reference Condition Approach predictive modeling showed a suggestion of a directional change in community structure over time. Only 1 of 15 reference sites that were repeatedly sampled over several years showed a pattern that may indicate a response to changing climate. Three, not mutually exclusive, reasons why we did not see a clear effect of climate change on BC stream ecosystems were: 1) Little or no effect of climate change relative to other, potentially interacting biotic and abiotic factors, 2) The timespan of monitoring was too short to detect cumulative effects of climate change, and, most importantly, 3) The sampling design and protocol were unable to detect climate change effects. To better detect and characterize the effects of climate change on streams in monitoring programs, we recommend annual re-sampling of a few reference sites and detailed analysis of the natural and human environment of the sites along with better characterization of the benthic community (e.g. with eDNA) at all monitored sites.

## Introduction

Human-caused climate change over the last 100 years is a scientifically accepted reality of the Anthropocene, and the prospect of an accelerated change of annual average as well as among- and within-year variation in precipitation and temperature has been extensively modeled.^1^ Climate change has had documented direct and indirect effects on freshwater ecosystems, particularly in boreal and temperate biomes, both on flow^2^ and biota.^3^ The direct and indirect effects of climate change on streams due to drought, unpredictable and extreme flow events, and changes in water temperature, among other factors, are predicted to increase over the next several decades.^4,5^

As in many jurisdictions around the world, including the UK, Australia, and the EU,^6^ Canada has a long-standing national biomonitoring program to assess the status of freshwater ecosystems exposed to various types of human activity. The Canadian Aquatic Biomonitoring Network (CABIN) program was first established in the 1990s and includes standard field,^7^ laboratory,^8^ and analysis protocols for using the Reference Condition Approach (RCA)^9^ to bioassessment in particular regions.^10^ The RCA relies on the development of a predictive model based on the relationship between reference sites (minimally exposed to human activity) and their natural environment, and application of that model to predict the biota at a test site (exposed to human activity) and assess whether or not test sites are in reference condition.

**Figure 1.**
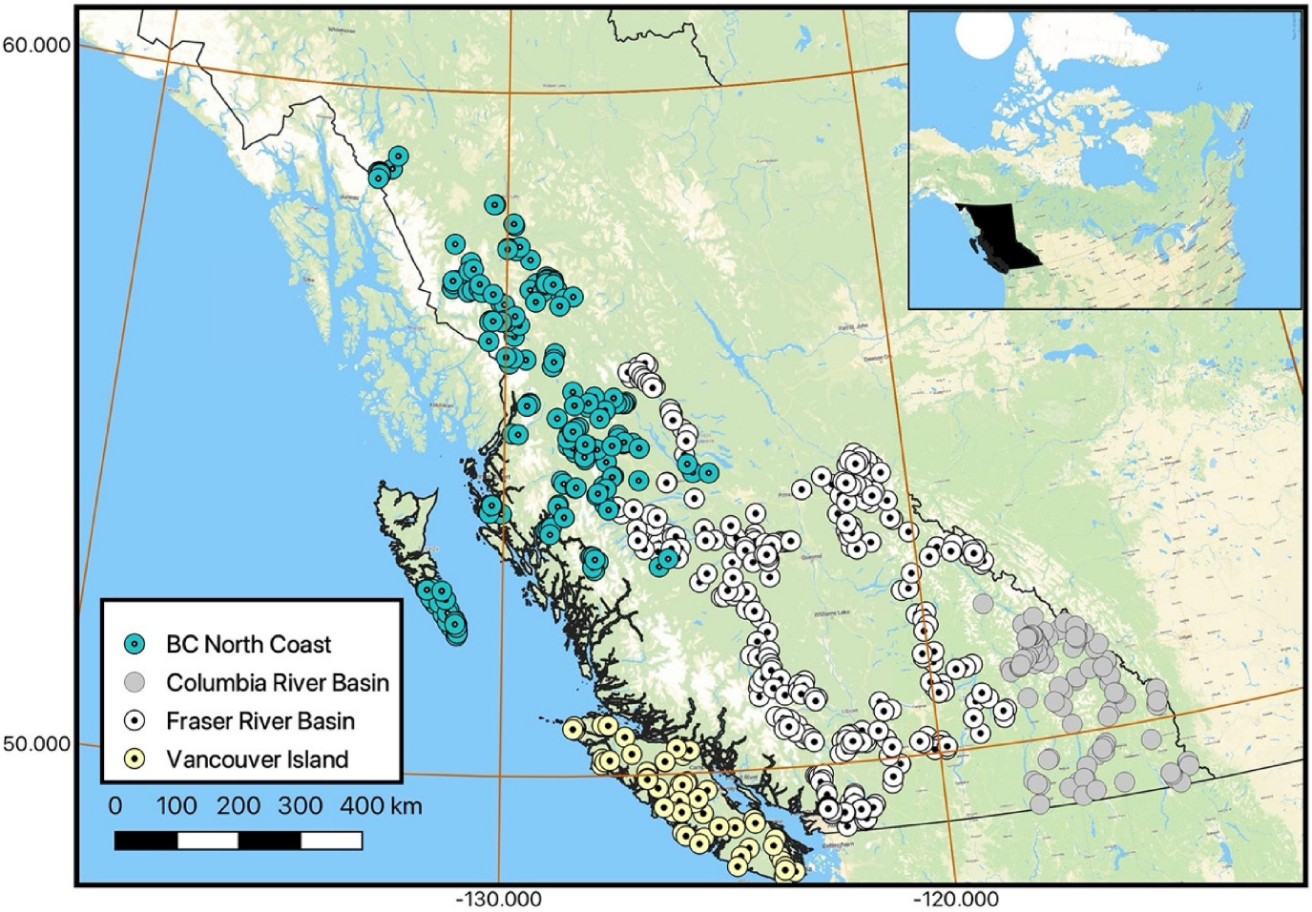
BC CABIN reference sites sampled between 1994 and 2021.

Datasets from long-term monitoring programs like CABIN may be valuable for assessing the effects of large spatial and temporal scale stressors like climate change. The purpose of this study was to determine if data collected from CABIN reference sites between 1994 and 2021 in British Columbia, Canada (Figure 1) showed any evidence of a response to climate change in benthic invertebrate stream assemblages. The data have been collected from seven regions across the province, but four, regional predictive models with a total of over 700 reference sites were analysed for this study (Table 1).

**Table 1.** Number of CABIN reference sites and sampling timespan in each BC CABIN region.

CABIN Region	# Reference Sites	Initial Sampling Year	Most Recent Sampling Year
BC North Coast	210	2004	2021
Columbia River Basin	127	2003	2018
Fraser River Basin	313	1994	2019
Vancouver Island	79	2001	2019
Total	729	1994	2021

## Methods

### Quantifying climate change at BC CABIN reference sites

There are many descriptors of climate (and thus, over time, climate change), including decadal average annual temperature, precipitation, and degree days. Historical air temperature and precipitation data from hundreds of weather stations across Canada have been used to create a 50 × 50 km grid of temperature and precipitation trends between 1948 and the 2010s.^11^ For temperature, the ^o^C change in annual mean temperature between 1948 and 2016 relative to the 1961–1990 baseline mean was determined for each grid square in Canada. For precipitation, the percentage change in total precipitation from 1948 to 2012 relative to the baseline mean was calculated for the same set of grid squares. We used GIS with these grids and the location of BC CABIN reference sites to determine temperature and precipitation trends over the last 70+ years at each site and then did a principal components analysis (PCA) of the correlation matrix of temperature and precipitation change to quantify a climate change index for each of the 100 s of CABIN sites.

It is important to distinguish the climate change index calculated for each reference site from precipitation and air temperature records over the particular period that the reference site was sampled. Our study was not concerned with the correlation between the benthic community of reference streams and the weather in the years or weeks immediately prior to stream sampling. Instead, we characterized the long-term climate change in a given area with the climate change index and then looked for correlated, directional, medium-term changes in the benthic community.

### Collecting and describing the benthic invertebrate community

Benthic invertebrate collections were made according to the CABIN field sampling protocol,^7^ including a three-minute traveling kick sample in a riffle habitat with a 400 µm mesh net followed by field preservation with formalin. Field samples were subsampled to 300 organisms and identified to Family level as documented in CABIN laboratory protocol.^8^

We used two methods to describe the diversity of the benthic invertebrate community in BC reference streams and how it varied in space and time:

- Alpha diversity of the community, the number of taxa found (i.e. taxon richness) at a site on a particular date.
- Beta diversity – the number of taxa found across all sites in each region in a particular sampling time period.

Increases or decreases in biodiversity are one potential effect of climate change, whereas more subtle changes in community structure (e.g. occurrence or proportion of different taxa) will often occur either in advance of or associated with biodiversity loss or gain. “Community structure” is by definition multivariate data since it is the number (or relative number) of individuals in each taxon present in a community. We compared community structure using two methods:

- Examining the pattern of occurrence of specific taxa among time periods within each region
- Using a distance measure to describe compositional variation among sites or a given site sampled multiple times. The distance measure summarizes with one number the differences in any two communities across all of the taxa found in both. We used Bray-Curtis Distance (BCDist) to compare pairs of communities and generate a distance matrix that summarizes these pairwise comparisons for a group of benthic communities, and Non-metric Multidimensional Scaling (NMDS) to efficiently describe the structure of a distance matrix in a two-dimensional scatter plot.

### Temporal dynamics of biota in BC reference streams

**Figure 2.**
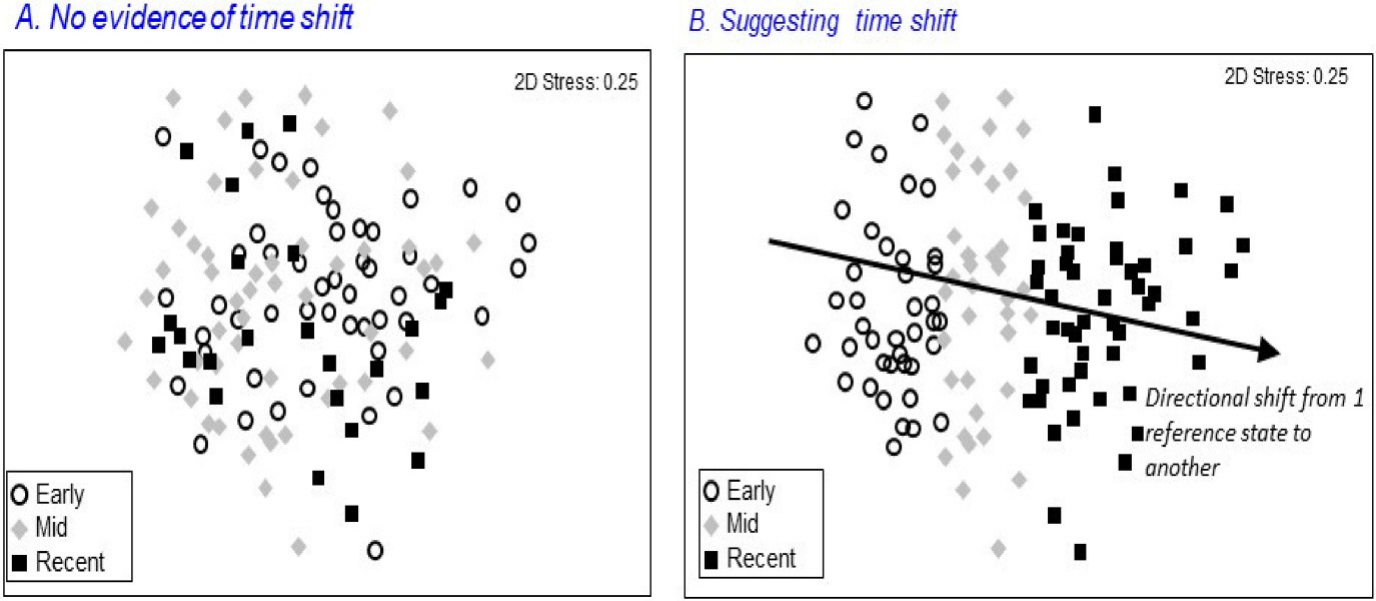
NMDS ordinations of a hypothetical group of reference sites either not showing (a) or showing (b) evidence of a directional shift in community structure over time consistent with an effect of climate change.

For each region, we compared taxon richness and occurrence among sites sampled in different time periods to look for change over time in either median or variability of taxon richness, and thus evidence of the effects of climate change on the community in a given region. Because of the sampling design in the different CABIN regional programs, we aggregated sampling years into half-decade periods. Depending on the starting and ending years of a program, some half-decade periods (e.g. 2021–2025) encompassed less than 5 years of sampling. We next examined temporal change in the community composition of BC reference sites in each BC region by comparing structure across time periods, but also looking more carefully at the trajectories of a small number of sites that were re-sampled over several years. As part of the CABIN RCA modeling process, reference sites are separated into faunally similar groups that are associated with a particular natural environment based on the CABIN predictive model.^10^ We presumed that if there was a response in a reference group associated with a long-term change in conditions, such as a climate shift, we would see a directional temporal change in community structure (Figure 2). For reference sites sampled repeatedly over a number of years (“trajectory sites”), we examined structural changes over time against variability among sites in its reference group.

## Results

### Climate change at BC CABIN reference sites

**Figure 3.**
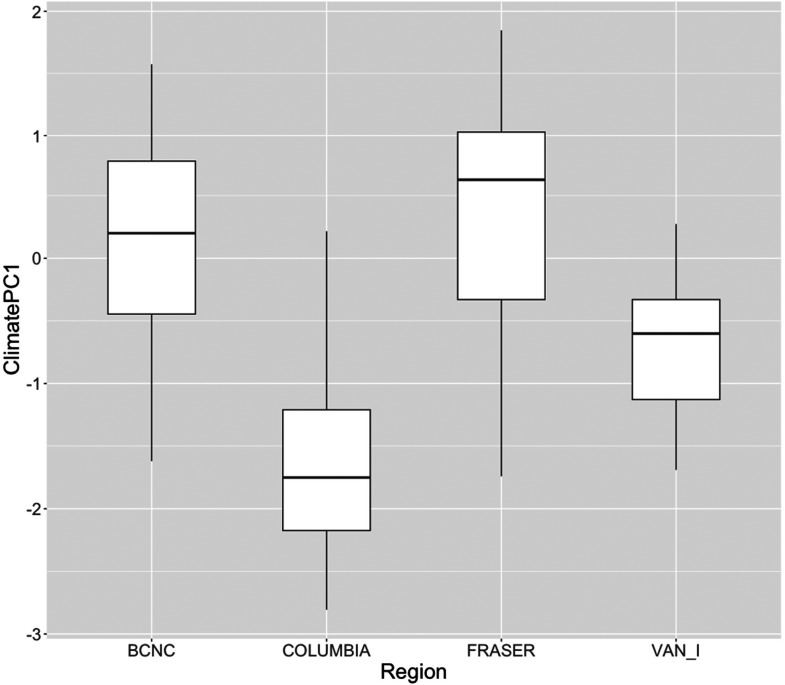
Variation among CABIN models in a major gradient of climate change (Climate PC1: increased temperature and decreased precipitation).

Based on historical data and trend modeling from ECCC,^11^ reference sites in all four CABIN regions showed a trend of increasing temperature from the mid-twentieth century to the 2010s. BC North Coast and Fraser River Basin had increases of between 1.75°C and 2°C over 60+ years, while the Columbia River Basin and Vancouver Island sites had more modest increases of between 1.25°C and 1.5°C. Precipitation trends over the same period broke down into two categories. The Fraser River Basin and Vancouver Island show little change in precipitation, although as with temperature, there is substantial variation among Fraser River Basin sites. The BC North Coast and Columbia River Basin sites show median trends of a 5–10% increase in annual precipitation. A Principal Component Analysis (PCA) of the correlation matrix of temperature and precipitation trends at BC reference sites across all datasets showed the main gradient of climate change among BC CABIN sites has been from a modest temperature increase and increased precipitation to greater temperature increase and a decline in precipitation. Comparing BC CABIN regions (Figure 3) showed evidence of a warmer and drier climate in BC North Coast and Fraser River Basin, while the Columbia River Basin and Vancouver Island areas show less of a warming, drying trend, and thus more modest climate change, through time.

### Spatial and temporal patterns in benthic invertebrate communities at BC CABIN reference sites

**Figure 4.**
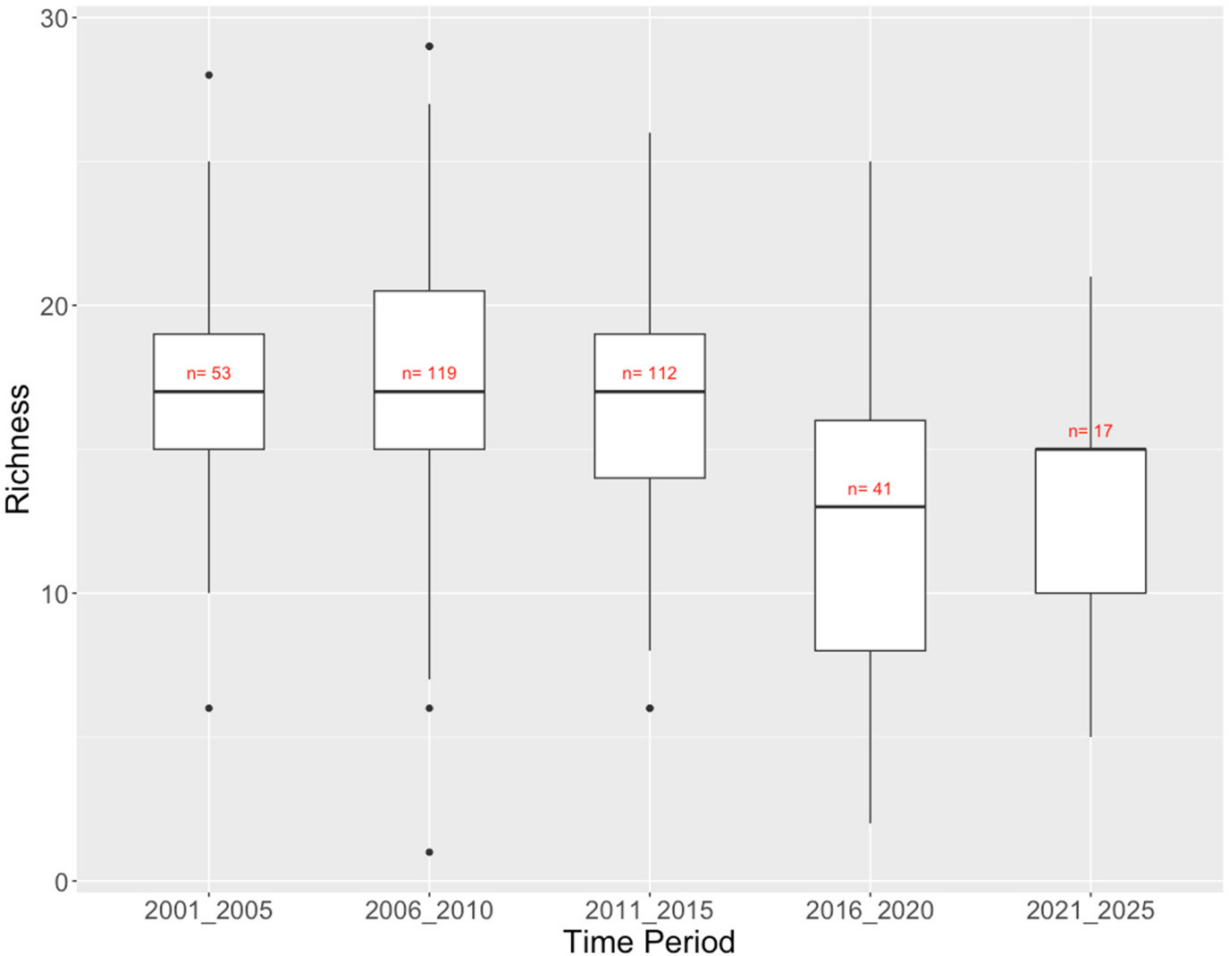
BC North Coast CABIN reference sites: richness (number of taxa) of benthic invertebrate communities by time period.

The BC North Coast dataset consists of 342 reference site samples covering 18 years from 2004 to 2021. Ninety-nine sites have been visited on more than one occasion, but only four have been visited in more than three sampling years and 79 of the repeated samples only have two records. There was substantial variation in taxon richness within every 5 years, with a notable drop in median richness over the last 10 years that is confounded by a drop in sampling effort from over 100 sites in 2006–2010 and 2011–2015 to 41 in 2016–2020 and only 17 in 2021–2025 (Figure 4). Total richness across all sites varied from 39 taxa in 2021–2025 to 69 taxa in 2011–2015, again tracking sampling effort. About half of the 89 taxa observed across all time periods were found in at least 4 of the 5 half-decades (Supplemental Table 1).

**Figure 5.**
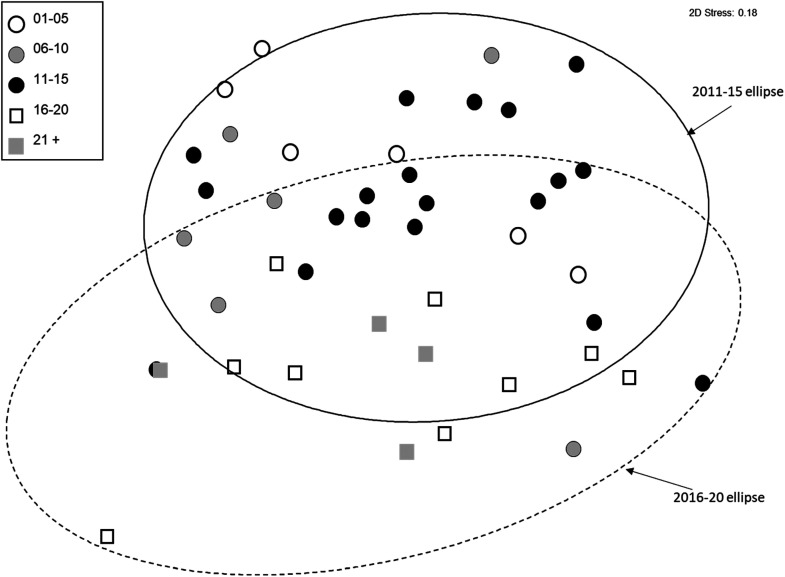
NMDS ordination of reference Group 4 from the North Coast BC. The 90% confidence ellipses of individual sites, which presume a normal distribution of ordination scores within the group, are shown for Group 4 sites sampled in the 2011–2015 and 2016–2020 periods.

The current RCA model for this region^12^ identifies five, faunally similar groups of reference sites. Four of these five groups showed little or no pattern of change in structure through time, while Group 4 (high altitude sites with low total abundance dominated by Heptageniidae mayflies and winter stoneflies) did show some evidence of temporal change from 2011–2015 to 2016–2020, but even this was quite modest (Figure 5). Only four of the BC North Coast sites had seven or more repeated visits over 15+ years. Although all showed temporal variability in structure, none showed a consistent, directional change over the time period sampled.

**Figure 6.**
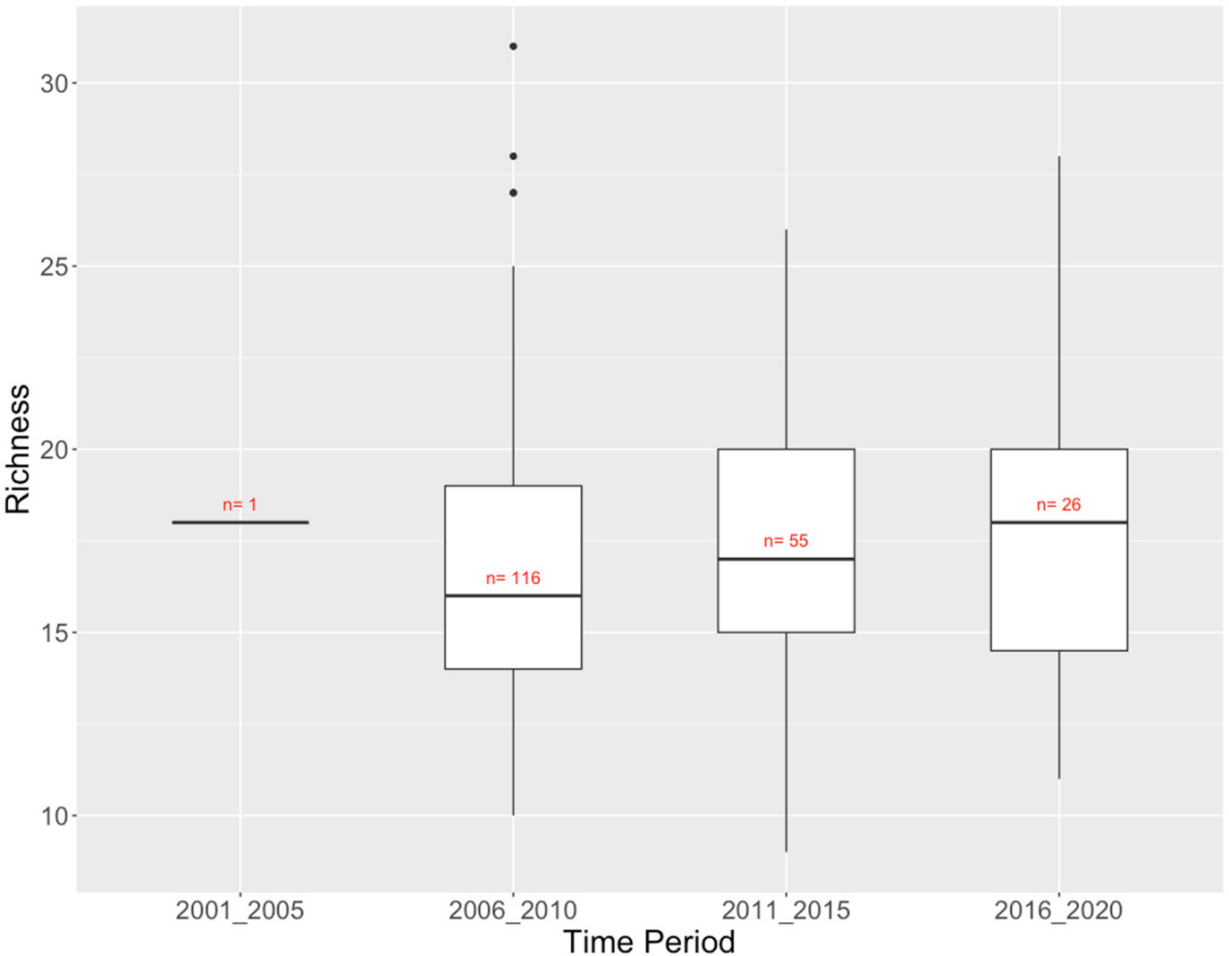
Columbia River Basin CABIN reference sites: richness (number of taxa) of benthic invertebrate communities by time period.

The Columbia River Basin dataset covers a 16-year period (2003–2018) and has 198 site samples in the CABIN model which identified six different reference groups.^13^ There was a modest trend of increasing richness at reference sites over the last 15 years (Figure 6). Total richness varied from 53 taxa in 2001–2005 (with only one site) to 62 taxa in 2006–2010, more or less tracking sampling effort. More than half of the 69 taxa observed across all time periods were found in at least 3 of the 4 half-decades (Supplementary Table 2).

**Figure 7.**
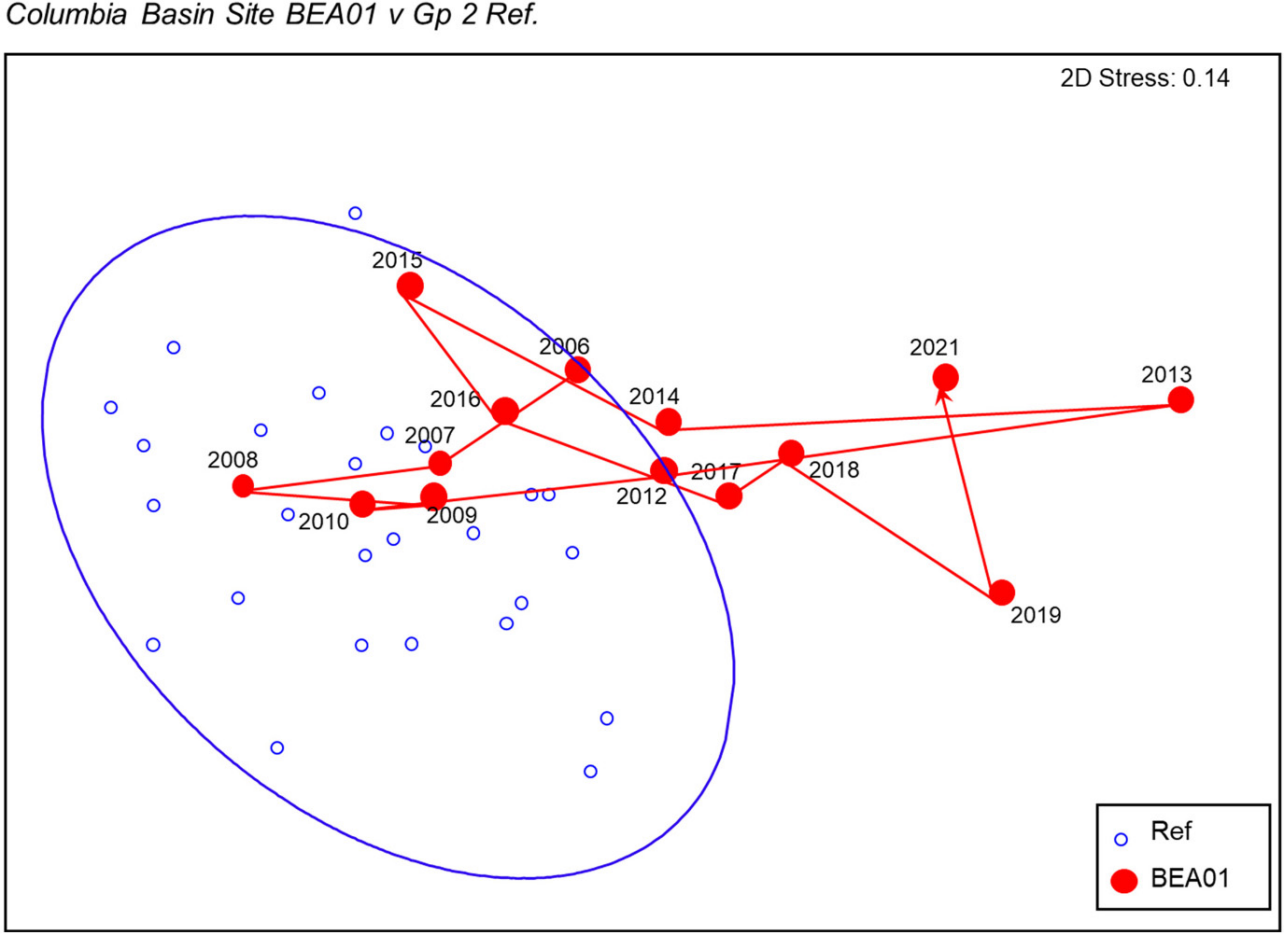
Trajectory of reference site BEA01 relative to the matched reference group from the Columbia River Basin. The 90% confidence ellipses of individual sites, which presume a normal distribution of ordination scores within the group, are shown for all Group 2 samples.

Four of the six reference groups were analyzed to determine if there was evidence of a long-term and consistent shift in community structure, but no discernible temporal pattern was observed. For the repeatedly sampled, trajectory sites, most never left or quickly returned to their reference group if they went outside it for a year or two. Site BEA01 does show evidence of a directional shift away from reference that is consistent with climate change effects (Figure 7). It is from reference Group 2 of the Columbia River Basin model, sites with relatively low total abundance dominated by Heptageniidae and Baetidae mayflies.

**Figure 8.**
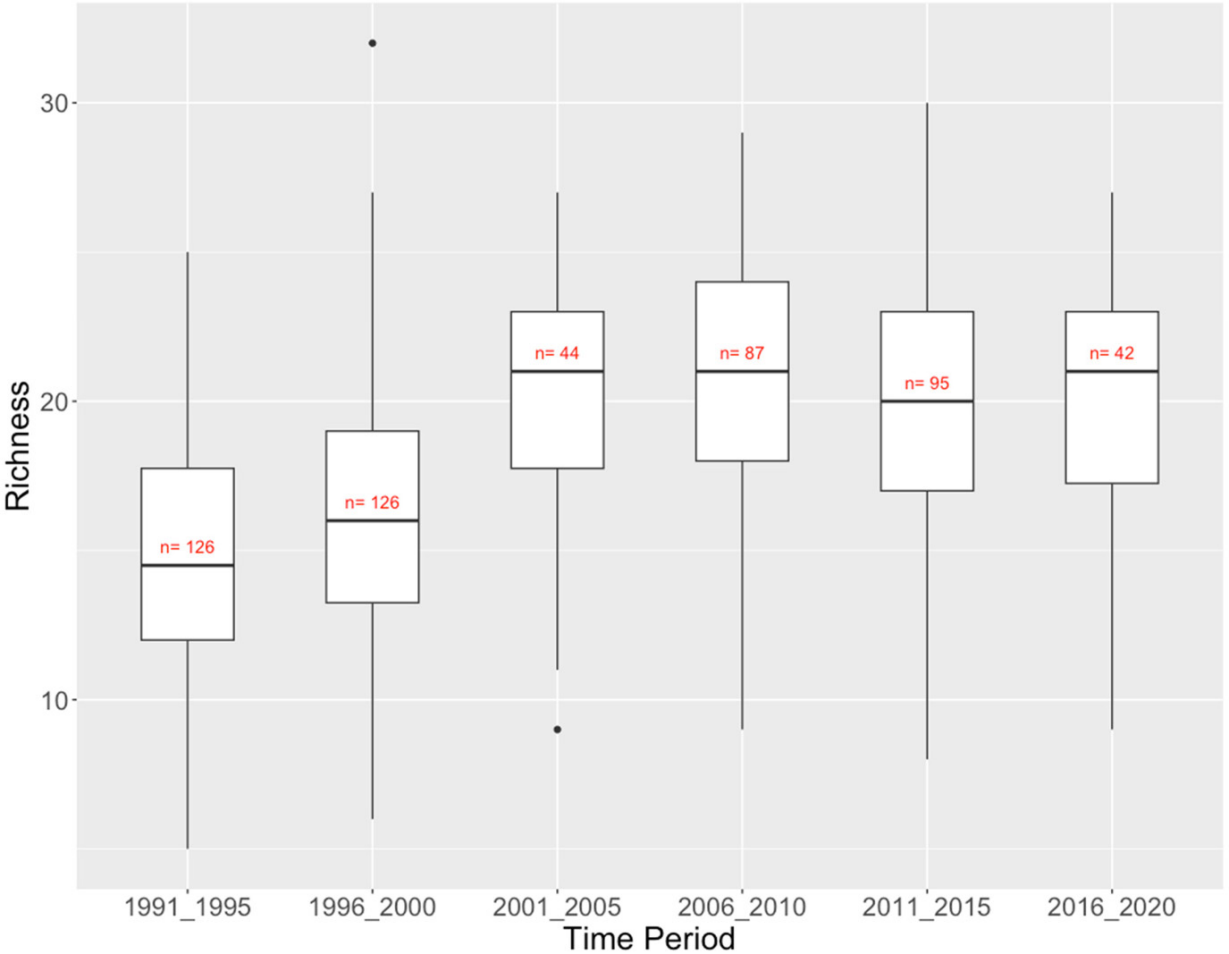
Fraser River Basin CABIN Reference Sites: Richness (number of taxa) of benthic invertebrate communities by time period.

The Fraser River Basin is the longest-running dataset, with samples from 1994 to 2019. It consists of 520 reference site samples, 274 of them being repeated visits to the same 63 sites. There was substantial variation in taxon richness within every five years, with richness increasing about 50% over the more than 25 years of sampling (Figure 8). Total richness across all sites varied from 53 taxa in 2016–2020 to 74 taxa in 1996–2000, with a weaker correlation with sampling effort than other regions. More than half of the 100 taxa observed across all time periods were found in at least 4 of the 6 half-decades. Only 8 of 39 taxa that were only found in two or fewer half-decades were observed in 2011–2015 or 2016–2020, while more than half of these less frequently observed taxa were found in the first 10 years of monitoring (1991–1995 and 1996–2000; Supplementary Table 3). There are 13 taxa that only occurred in one of the early (1991–1995, 1996–2000) time periods, including Asellidae, Glossiphoniidae, Haliplidae, and Margaritiferidae. There were only three taxa that occurred in one of the late (2011–2015, 2016–2020) time periods: Corixidae, Dryopidae, and Psychomyiidae.

Six reference groups have been identified in the Fraser River Basin's current CABIN model,^10^ and none of the groups showed more than a very modest temporal change in community structure over the full range of years sampled. The site with the longest period of record, with 19 sampling years covering a span of 27 years, is on the 5th-order Coldwater River (CLD01), a tributary of the Thompson River. It has usually been classified in Group 4a, which is dominated by Heptageniidae, Chironomidae, Baetidae and Ephemerellidae and mostly higher altitude streams with relatively small drainage areas.^10^ When first sampled (1995–1996), CLD01 was on the periphery of its reference group and remained there for several years. In 2004–2005, CLD01 moved closer to reference, and in 2006 it moved well out of reference. Analysis of the benthic invertebrate abundances showed this was a result of a decline in total abundance, particularly a loss of Ephemerellidae and Lepidostomatidae. In 2007, CLD01 moved back toward reference and in 2008 it is almost in the center of the reference cloud. Again in 2009 and 2010, CLD01 showed a shift out of reference, and in the period 2012–2019 it is back in reference condition. It is only in 2021 that a big shift away from the reference condition is seen, which has been attributed to the extensive forest fires in the period before sampling (S. Strachan; pers. comm.), an indirect effect of climate change.

**Figure 9.**
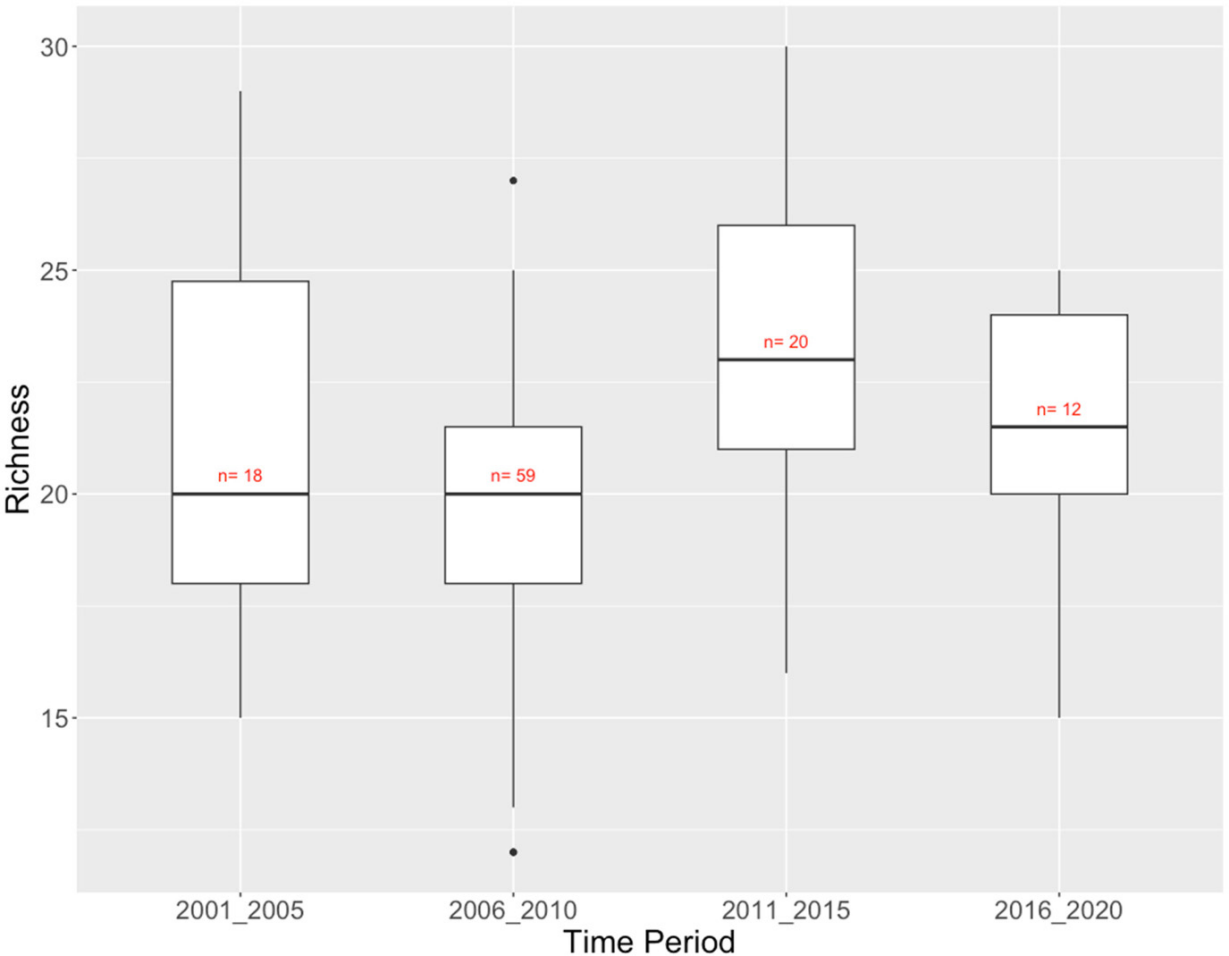
Vancouver Island CABIN reference sites: richness (number of taxa) of benthic invertebrate communities by time period.

The Vancouver Island dataset consists of only 109 reference sites sampled over a period of 19 years (2001–2019). There was a modest upward trend in richness over the 19-year sampling period (Figure 9). Total richness was relatively constant among time periods, varying from 48 taxa in 2016–2020 to 54 taxa in 2006–2010, which was also the half-decade with by far the greatest sampling effort (59 sites versus 20 at most in the other half-decades). More than half of the 74 taxa observed across all time periods were found in all 4 half-decades sampled (Supplementary Table 4).

**Figure 10.**
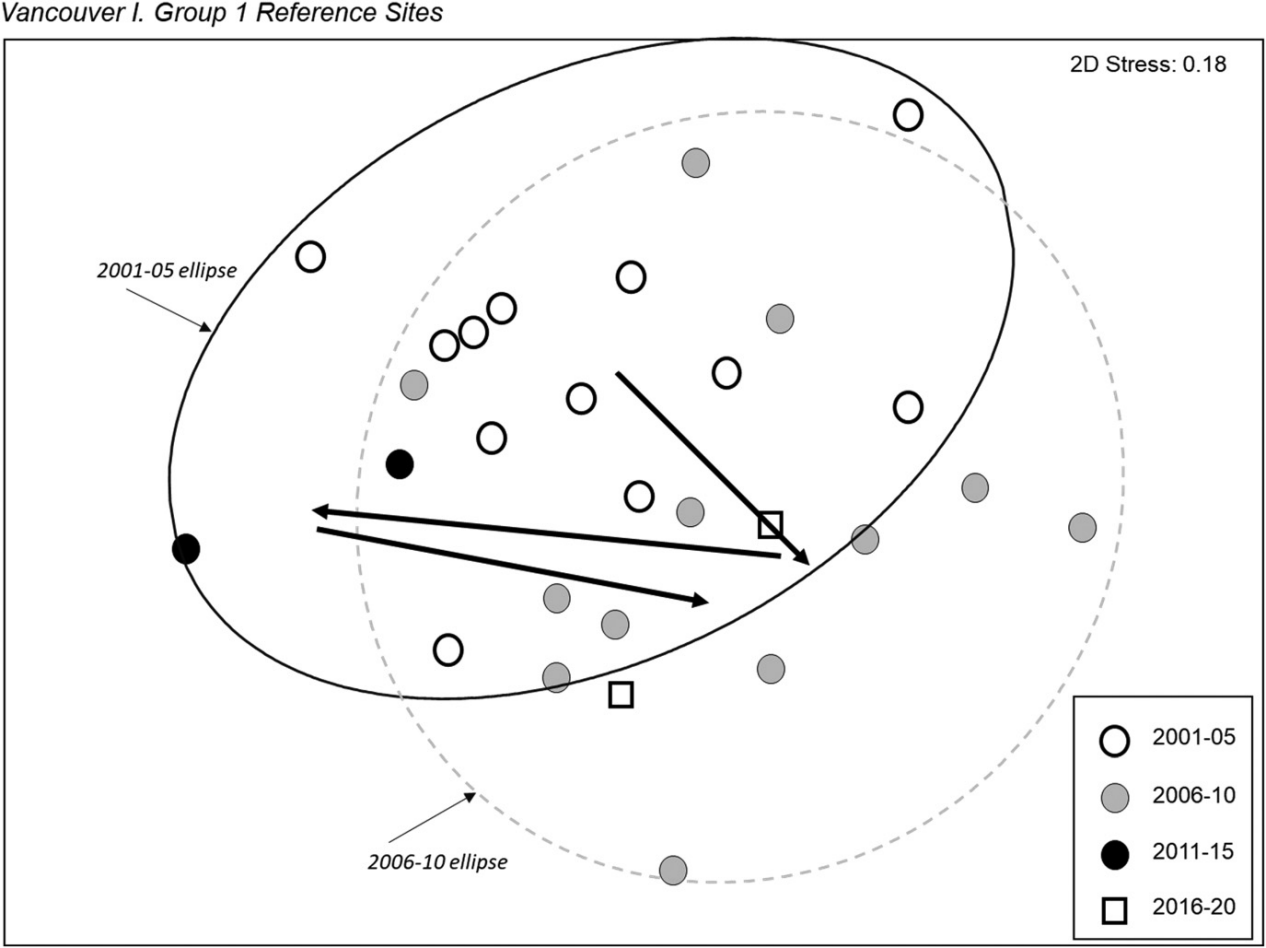
NMDS ordination of reference Group 1 from Vancouver Island. The 90% confidence ellipses of individual sites, which presume a normal distribution of ordination scores within the group, are shown for Group 1 sites sampled in the 2001–2005 and 2006–2010 time periods.

**Figure 11.**
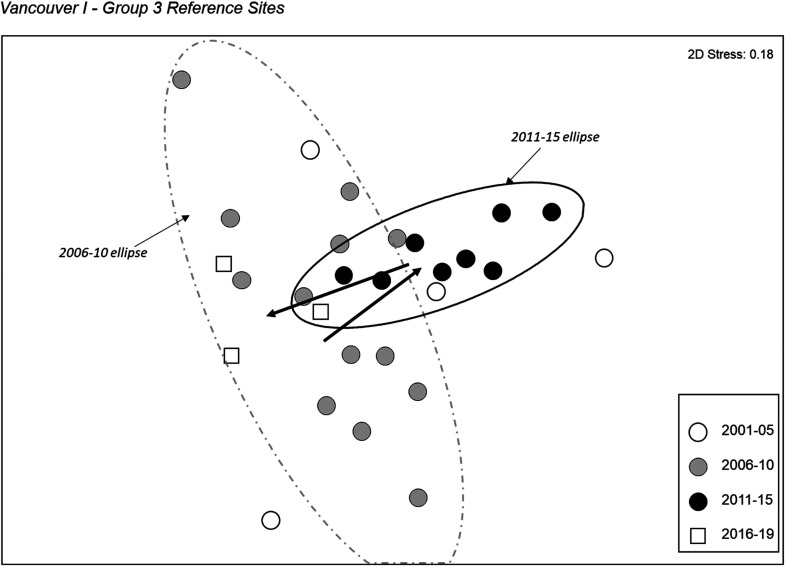
NMDS ordination of reference Group 3 from Vancouver Island. The 90% confidence ellipses of individual sites, which presume a normal distribution of ordination scores within the group, are shown for Group 3 sites sampled in the 2006–2010 and 2011–2015 time periods.

Three of the four reference groups in the Vancouver Island dataset that were identified in the current model^14^ and had sufficient sites for analysis were examined for a change in reference condition through time. Group 4 sites show no evidence of a change in the community assemblage through time, however, both Group 1 (Figure 10) and Group 3 (Figure 11) sites show a distinct shift from the earliest time period sampled with respect to both the median and variability of community structure. Two trajectory sites (NIAG-01, TSUL-01) with 7 or more sampling years were examined individually. Both sites showed substantial temporal variability but no clear directional change in community structure.

## Discussion

Across all four regions in British Columbia, Canada, that has a long-term dataset collected using the CABIN monitoring protocol, the Fraser River Basin reference sites showed a distinct trend of increasing alpha biodiversity across the sampling time span, while the Columbia River Basin and Vancouver Island sites showed a more modest trend of increasing richness and the BC North Coast showed a modest trend of decreasing taxon richness. There was some variability in total taxon richness among half-decades in each region (Tables 2–5), but this seemed primarily due to the number of sites sampled within the period (i.e. sampling effort).

Increasing alpha diversity in Fraser River reference sites over the 25 years of sampling may reflect climate change effects. The warmer and drier climate in the region may enable a greater diversity of taxa at a stream site because of a change in the variety, amount, or within-year variability of habitat and food resources available.

About 50% or more of the taxa ever found in a region occurred over most of the half-decadal periods (Tables 2–5), indicating little evidence of losses or gains of taxa at a regional scale over the timespan of monitoring that may indicate a climate change signal. The exception to this is, again, the Fraser River Basin sites. Although alpha diversity increased over time at the Fraser River Basin reference sites (Figure 8), taxa that were only found in one or two half-decades were mostly (31/39) found in the first 10 years of the 25-year monitoring period (Supplementary Table 3). This is evidence that although each reference site can accommodate more taxa (i.e. alpha diversity is higher) there are fewer taxa “available” to reference sites in later years.

Only three of the 21 reference groups (defined in CABIN modeling) showed any evidence of a shift in community structure through 5-year time periods. For resampled, “trajectory” sites, just 1 of 15 sites (BEA01 in the Columbia River Basin) showed a directional change in community structure that may indicate a response to changing climate.

Analysis of over 700 reference streams in British Columbia sampled over more than 25 years showed only modest evidence of what may be a change in the biota in response to climate change. Because of the nature of the CABIN datasets, there are three likely, and not mutually exclusive, reasons for this result:

1. **There is little or no effect of climate change on biota in most BC streams –** it is possible that the degree and nature of climate change in BC stream ecosystems where CABIN sampling was carried out did not cause substantive changes in the stream benthic community over the 25+ years the CABIN program has been executed in BC. This seems highly unlikely given well-documented evidence of the direct and indirect effects of a changing climate on temperate and boreal stream ecosystems e.g.,^1–3^
2. **The CABIN sampling period was too short to detect climate change effects on most BC streams** Small effects on the benthic community may not have sufficiently accumulated over the sampling periods examined to be evident. Detection of so-called cumulative effects is a vexing problem in most if not all bioassessment and biomonitoring programs.^15^ Again, this seems unlikely, particularly for regions like the Fraser River Basin that have been monitored for more than 20 years.
3. **The CABIN sampling protocol is unable to detect climate change effects on biota in most BC streams –** The application of the Reference Condition Approach^9^ with CABIN field^7^ and laboratory^8^ protocols was designed to distinguish sites that are in reference condition from those that are out of reference. Although the protocol has been relatively successful in distinguishing sites in reference condition from those that have been degraded by human activity, the accuracy and precision of this sampling protocol may be insufficient to detect the year-to-year subtler changes in the community in response to a changing climate. In particular, estimating biodiversity and community structure based only on morphological identification of taxa may be too crude. The lack of consistent long-term sampling of at least a subset of sites limits the ability to detect temporal patterns, and there is sampling bias and imprecision at every step of the protocol from the kick sample collection, to Marchant Box subsampling, to identification to mainly Family-level resolution using only morphological traits. The greatly reduced cost of obtaining more accurate and precise estimates from eDNA analysis^16–18^ suggests that it should become part of the standard protocol of large-scale monitoring programs for two reasons: increased sensitivity to change, including climate change effects, and better estimates of the biodiversity present in systems that are vulnerable to change before we lose it. Other types of biological information, including traits of the biota^19^ or fatty acids,^20^ may also help inform the diagnosis of why an ecosystem is not in reference condition. Many of these, including trait-based analyses in particular, require precise and accurate identification of taxa with resolution greater than that possible with morphological identification.

In addition to better descriptions of the biota, more comprehensive descriptions of the natural^21^ and human activity^22^ environments will produce better models predicting the biota from the natural environment and diagnosing the causes of deviations from reference conditions including climate change. There are readily available GIS tools and remote sensing data, including satellite images, to support this work.

To address the challenges in continuing to use the RCA in efficient and effective biomonitoring, but also being mindful of changes in the reference condition because of climate change and other factors, we recommend annually sampling a small subset of reference streams in each predictive model region to assess year-to-year variability and directional changes in the community. We also strongly recommend improved characterization of the biota with eDNA sampling to better characterize biodiversity and support traits-based analyses. With respect to assessing the effects of climate change and other long-term and large spatial-scale stressors, archived datasets like those maintained by the Canadian CABIN program are invaluable in indicating specific ecosystems or regions that show response to a changing climate. Further analysis with available historical and traditional knowledge about stream ecosystems of particular interest can elucidate the direct and indirect effects of climate change on stream ecosystems.

## Supplemental Material

sj-docx-1-sci-10.1177_00368504231219335 - Supplemental material for Can datasets from long-term biomonitoring programs detect climate change effects on stream benthos? Click here for additional data file. Supplemental material, sj-docx-1-sci-10.1177_00368504231219335 for Can datasets from long-term biomonitoring programs detect climate change effects on stream benthos? by Robert C. Bailey and Trefor B. Reynoldson in Science Progress
