# Supplementary material for: Can datasets from long-term biomonitoring programs detect climate change effects on stream benthos?
Source: Sci Prog. 2023 Dec 17;106(4):00368504231219335. doi: 10.1177/00368504231219335 (PMC10729632; doi:10.1177/00368504231219335)
Supplement: sj-docx-1-sci-10.1177_00368504231219335 - Supplemental material for Can datasets from long-term biomonitoring programs detect climate change effects on stream benthos? [file sj-docx-1-sci-10.1177_00368504231219335.docx]

**Supplementary Table 1** – BC North Coast Taxon Occurrence at Reference Sites

| **Taxon** | **2001_2005** | **2006_2010** | **2011_2015** | **2016_2020** | **2021_2025** | **nOccur** |
| --- | --- | --- | --- | --- | --- | --- |
| Ameletidae | 1 | 1 | 1 | 1 | 1 | 5 |
| Baetidae | 1 | 1 | 1 | 1 | 1 | 5 |
| Brachycentridae | 1 | 1 | 1 | 1 | 1 | 5 |
| Capniidae | 1 | 1 | 1 | 1 | 1 | 5 |
| Chironomidae | 1 | 1 | 1 | 1 | 1 | 5 |
| Chloroperlidae | 1 | 1 | 1 | 1 | 1 | 5 |
| Elmidae | 1 | 1 | 1 | 1 | 1 | 5 |
| Empididae | 1 | 1 | 1 | 1 | 1 | 5 |
| Enchytraeidae | 1 | 1 | 1 | 1 | 1 | 5 |
| Ephemerellidae | 1 | 1 | 1 | 1 | 1 | 5 |
| Glossosomatidae | 1 | 1 | 1 | 1 | 1 | 5 |
| Heptageniidae | 1 | 1 | 1 | 1 | 1 | 5 |
| Hydropsychidae | 1 | 1 | 1 | 1 | 1 | 5 |
| Hydryphantidae | 1 | 1 | 1 | 1 | 1 | 5 |
| Hygrobatidae | 1 | 1 | 1 | 1 | 1 | 5 |
| Lebertiidae | 1 | 1 | 1 | 1 | 1 | 5 |
| Leptophlebiidae | 1 | 1 | 1 | 1 | 1 | 5 |
| Leuctridae | 1 | 1 | 1 | 1 | 1 | 5 |
| Limnephilidae | 1 | 1 | 1 | 1 | 1 | 5 |
| Naididae | 1 | 1 | 1 | 1 | 1 | 5 |
| Nemouridae | 1 | 1 | 1 | 1 | 1 | 5 |
| Perlodidae | 1 | 1 | 1 | 1 | 1 | 5 |
| Rhyacophilidae | 1 | 1 | 1 | 1 | 1 | 5 |
| Simuliidae | 1 | 1 | 1 | 1 | 1 | 5 |
| Sperchontidae | 1 | 1 | 1 | 1 | 1 | 5 |
| Taeniopterygidae | 1 | 1 | 1 | 1 | 1 | 5 |
| Tipulidae | 1 | 1 | 1 | 1 | 1 | 5 |
| Torrenticolidae | 1 | 1 | 1 | 1 | 1 | 5 |
| Uenoidae | 1 | 1 | 1 | 1 | 1 | 5 |
| Apataniidae | 1 | 1 | 1 | 0 | 1 | 4 |
| Blephariceridae | 1 | 1 | 1 | 1 | 0 | 4 |
| Ceratopogonidae | 1 | 1 | 1 | 1 | 0 | 4 |
| Deuterophlebiidae | 1 | 1 | 1 | 1 | 0 | 4 |
| Dytiscidae | 1 | 1 | 1 | 1 | 0 | 4 |
| Hydroptilidae | 1 | 1 | 1 | 0 | 1 | 4 |
| Hydrozetidae | 0 | 1 | 1 | 1 | 1 | 4 |
| Lepidostomatidae | 1 | 1 | 1 | 1 | 0 | 4 |
| Lumbriculidae | 1 | 1 | 1 | 1 | 0 | 4 |
| Perlidae | 1 | 1 | 1 | 1 | 0 | 4 |
| Philopotamidae | 1 | 1 | 1 | 1 | 0 | 4 |
| Psychodidae | 1 | 1 | 1 | 1 | 0 | 4 |
| Aturidae | 0 | 1 | 1 | 0 | 1 | 3 |
| Curculionidae | 1 | 1 | 1 | 0 | 0 | 3 |
| Ephydridae | 1 | 1 | 1 | 0 | 0 | 3 |
| Hydrophilidae | 1 | 1 | 0 | 1 | 0 | 3 |
| Isotomidae | 1 | 1 | 1 | 0 | 0 | 3 |
| Lumbricidae | 0 | 0 | 1 | 1 | 1 | 3 |
| Pisidiidae | 1 | 1 | 1 | 0 | 0 | 3 |
| Planorbidae | 0 | 1 | 1 | 1 | 0 | 3 |
| Sminthuridae | 1 | 1 | 0 | 0 | 1 | 3 |
| Staphylinidae | 1 | 0 | 1 | 1 | 0 | 3 |
| Valvatidae | 1 | 1 | 1 | 0 | 0 | 3 |
| Aphididae | 1 | 1 | 0 | 0 | 0 | 2 |
| Athericidae | 1 | 1 | 0 | 0 | 0 | 2 |
| Dixidae | 0 | 0 | 1 | 1 | 0 | 2 |
| Gammaridae | 0 | 1 | 1 | 0 | 0 | 2 |
| Hyalellidae | 0 | 1 | 1 | 0 | 0 | 2 |
| Leptoceridae | 0 | 1 | 1 | 0 | 0 | 2 |
| Lymnaeidae | 0 | 0 | 1 | 1 | 0 | 2 |
| Mideopsidae | 0 | 1 | 1 | 0 | 0 | 2 |
| Muscidae | 0 | 0 | 0 | 1 | 1 | 2 |
| Phoridae | 1 | 0 | 0 | 0 | 1 | 2 |
| Polycentropodidae | 0 | 1 | 1 | 0 | 0 | 2 |
| Sciomyzidae | 1 | 1 | 0 | 0 | 0 | 2 |
| Sphaeromatidae | 0 | 1 | 1 | 0 | 0 | 2 |
| Stygothrombiidae | 1 | 0 | 0 | 1 | 0 | 2 |
| Arrenuridae | 0 | 0 | 1 | 0 | 0 | 1 |
| Chaoboridae | 0 | 0 | 1 | 0 | 0 | 1 |
| Cicadellidae | 0 | 1 | 0 | 0 | 0 | 1 |
| Crangonyctidae | 0 | 1 | 0 | 0 | 0 | 1 |
| Dolichopodidae | 0 | 0 | 0 | 0 | 1 | 1 |
| Feltriidae | 0 | 0 | 1 | 0 | 0 | 1 |
| Glossiphoniidae | 0 | 0 | 1 | 0 | 0 | 1 |
| Gomphidae | 1 | 0 | 0 | 0 | 0 | 1 |
| Halacaridae | 1 | 0 | 0 | 0 | 0 | 1 |
| Haplotaxidae | 0 | 0 | 1 | 0 | 0 | 1 |
| Hydraenidae | 0 | 0 | 1 | 0 | 0 | 1 |
| Hydrobiidae | 0 | 0 | 1 | 0 | 0 | 1 |
| Limnoriidae | 0 | 1 | 0 | 0 | 0 | 1 |
| Limnozetidae | 0 | 0 | 0 | 0 | 1 | 1 |
| Mideidae | 0 | 0 | 1 | 0 | 0 | 1 |
| Oreoleptidae | 0 | 1 | 0 | 0 | 0 | 1 |
| Oxidae | 0 | 0 | 1 | 0 | 0 | 1 |
| Poduridae | 0 | 1 | 0 | 0 | 0 | 1 |
| Psephenidae | 0 | 0 | 1 | 0 | 0 | 1 |
| Pteronarcyidae | 1 | 0 | 0 | 0 | 0 | 1 |
| Sarcophagidae | 1 | 0 | 0 | 0 | 0 | 1 |
| Siphlonuridae | 0 | 0 | 1 | 0 | 0 | 1 |
| Stratiomyidae | 0 | 1 | 0 | 0 | 0 | 1 |
| **Total Diversity** | 57 | 65 | 69 | 47 | 39 |  |

**Supplementary Table 2** – Columbia River Basin Taxon Occurrence at Reference Sites

| **Taxon** | **2001_2005** | **2006_2010** | **2011_2015** | **2016_2020** | **nOccur** |
| --- | --- | --- | --- | --- | --- |
| Baetidae | 1 | 1 | 1 | 1 | 4 |
| Capniidae | 1 | 1 | 1 | 1 | 4 |
| Chironomidae | 1 | 1 | 1 | 1 | 4 |
| Chloroperlidae | 1 | 1 | 1 | 1 | 4 |
| Empididae | 1 | 1 | 1 | 1 | 4 |
| Enchytraeidae | 1 | 1 | 1 | 1 | 4 |
| Ephemerellidae | 1 | 1 | 1 | 1 | 4 |
| Heptageniidae | 1 | 1 | 1 | 1 | 4 |
| Hydropsychidae | 1 | 1 | 1 | 1 | 4 |
| Hygrobatidae | 1 | 1 | 1 | 1 | 4 |
| Lebertiidae | 1 | 1 | 1 | 1 | 4 |
| Lepidostomatidae | 1 | 1 | 1 | 1 | 4 |
| Perlidae | 1 | 1 | 1 | 1 | 4 |
| Perlodidae | 1 | 1 | 1 | 1 | 4 |
| Psychodidae | 1 | 1 | 1 | 1 | 4 |
| Sperchontidae | 1 | 1 | 1 | 1 | 4 |
| Taeniopterygidae | 1 | 1 | 1 | 1 | 4 |
| Ameletidae | 0 | 1 | 1 | 1 | 3 |
| Apataniidae | 0 | 1 | 1 | 1 | 3 |
| Brachycentridae | 0 | 1 | 1 | 1 | 3 |
| Ceratopogonidae | 0 | 1 | 1 | 1 | 3 |
| Elmidae | 0 | 1 | 1 | 1 | 3 |
| Glossosomatidae | 0 | 1 | 1 | 1 | 3 |
| Hydryphantidae | 0 | 1 | 1 | 1 | 3 |
| Leptophlebiidae | 0 | 1 | 1 | 1 | 3 |
| Leuctridae | 0 | 1 | 1 | 1 | 3 |
| Limnephilidae | 0 | 1 | 1 | 1 | 3 |
| Lumbriculidae | 0 | 1 | 1 | 1 | 3 |
| Naididae | 0 | 1 | 1 | 1 | 3 |
| Nemouridae | 0 | 1 | 1 | 1 | 3 |
| Peltoperlidae | 0 | 1 | 1 | 1 | 3 |
| Philopotamidae | 0 | 1 | 1 | 1 | 3 |
| Rhyacophilidae | 0 | 1 | 1 | 1 | 3 |
| Simuliidae | 0 | 1 | 1 | 1 | 3 |
| Tipulidae | 0 | 1 | 1 | 1 | 3 |
| Torrenticolidae | 0 | 1 | 1 | 1 | 3 |
| Uenoidae | 0 | 1 | 1 | 1 | 3 |
| Athericidae | 0 | 1 | 0 | 1 | 2 |
| Aturidae | 0 | 0 | 1 | 1 | 2 |
| Dytiscidae | 0 | 1 | 1 | 0 | 2 |
| Hydroptilidae | 0 | 1 | 1 | 0 | 2 |
| Hydrozetidae | 0 | 1 | 1 | 0 | 2 |
| Pisidiidae | 0 | 1 | 1 | 0 | 2 |
| Planariidae | 0 | 1 | 1 | 0 | 2 |
| Planorbidae | 0 | 1 | 1 | 0 | 2 |
| Stygothrombiidae | 0 | 1 | 0 | 1 | 2 |
| Tabanidae | 0 | 1 | 1 | 0 | 2 |
| Blephariceridae | 0 | 1 | 0 | 0 | 1 |
| Candonidae | 1 | 0 | 0 | 0 | 1 |
| Carabidae | 0 | 1 | 0 | 0 | 1 |
| Corixidae | 0 | 1 | 0 | 0 | 1 |
| Curculionidae | 0 | 0 | 1 | 0 | 1 |
| Deuterophlebiidae | 0 | 1 | 0 | 0 | 1 |
| Dixidae | 0 | 1 | 0 | 0 | 1 |
| Ephydridae | 0 | 1 | 0 | 0 | 1 |
| Feltriidae | 0 | 0 | 0 | 1 | 1 |
| Hydraenidae | 0 | 0 | 1 | 0 | 1 |
| Hydridae | 0 | 1 | 0 | 0 | 1 |
| Leptoceridae | 0 | 0 | 0 | 1 | 1 |
| Mideopsidae | 0 | 1 | 0 | 0 | 1 |
| Oreoleptidae | 0 | 1 | 0 | 0 | 1 |
| Pelecorhynchidae | 0 | 1 | 0 | 0 | 1 |
| Poduridae | 0 | 1 | 0 | 0 | 1 |
| Psephenidae | 0 | 0 | 1 | 0 | 1 |
| Pteronarcyidae | 0 | 1 | 0 | 0 | 1 |
| Sciomyzidae | 0 | 1 | 0 | 0 | 1 |
| Sminthuridae | 0 | 1 | 0 | 0 | 1 |
| Thaumaleidae | 0 | 1 | 0 | 0 | 1 |
| Valvatidae | 0 | 1 | 0 | 0 | 1 |
| **Total Diversity** | 18 | 62 | 48 | 42 |  |

**Supplementary Table 3** – Fraser River Basin Taxon Occurrence at Reference Sites

| **Taxon** | **1991_1995** | **1996_2000** | **2001_2005** | **2006_2010** | **2011_2015** | **2016_2020** | **nOccur** |
| --- | --- | --- | --- | --- | --- | --- | --- |
| Ameletidae | 1 | 1 | 1 | 1 | 1 | 1 | 6 |
| Apataniidae | 1 | 1 | 1 | 1 | 1 | 1 | 6 |
| Athericidae | 1 | 1 | 1 | 1 | 1 | 1 | 6 |
| Baetidae | 1 | 1 | 1 | 1 | 1 | 1 | 6 |
| Brachycentridae | 1 | 1 | 1 | 1 | 1 | 1 | 6 |
| Capniidae | 1 | 1 | 1 | 1 | 1 | 1 | 6 |
| Ceratopogonidae | 1 | 1 | 1 | 1 | 1 | 1 | 6 |
| Chironomidae | 1 | 1 | 1 | 1 | 1 | 1 | 6 |
| Chloroperlidae | 1 | 1 | 1 | 1 | 1 | 1 | 6 |
| Elmidae | 1 | 1 | 1 | 1 | 1 | 1 | 6 |
| Empididae | 1 | 1 | 1 | 1 | 1 | 1 | 6 |
| Enchytraeidae | 1 | 1 | 1 | 1 | 1 | 1 | 6 |
| Ephemerellidae | 1 | 1 | 1 | 1 | 1 | 1 | 6 |
| Glossosomatidae | 1 | 1 | 1 | 1 | 1 | 1 | 6 |
| Heptageniidae | 1 | 1 | 1 | 1 | 1 | 1 | 6 |
| Hydropsychidae | 1 | 1 | 1 | 1 | 1 | 1 | 6 |
| Hydroptilidae | 1 | 1 | 1 | 1 | 1 | 1 | 6 |
| Hydryphantidae | 1 | 1 | 1 | 1 | 1 | 1 | 6 |
| Lebertiidae | 1 | 1 | 1 | 1 | 1 | 1 | 6 |
| Lepidostomatidae | 1 | 1 | 1 | 1 | 1 | 1 | 6 |
| Leptoceridae | 1 | 1 | 1 | 1 | 1 | 1 | 6 |
| Leptophlebiidae | 1 | 1 | 1 | 1 | 1 | 1 | 6 |
| Leuctridae | 1 | 1 | 1 | 1 | 1 | 1 | 6 |
| Limnephilidae | 1 | 1 | 1 | 1 | 1 | 1 | 6 |
| Lumbriculidae | 1 | 1 | 1 | 1 | 1 | 1 | 6 |
| Naididae | 1 | 1 | 1 | 1 | 1 | 1 | 6 |
| Nemouridae | 1 | 1 | 1 | 1 | 1 | 1 | 6 |
| Perlidae | 1 | 1 | 1 | 1 | 1 | 1 | 6 |
| Perlodidae | 1 | 1 | 1 | 1 | 1 | 1 | 6 |
| Pisidiidae | 1 | 1 | 1 | 1 | 1 | 1 | 6 |
| Psychodidae | 1 | 1 | 1 | 1 | 1 | 1 | 6 |
| Pteronarcyidae | 1 | 1 | 1 | 1 | 1 | 1 | 6 |
| Rhyacophilidae | 1 | 1 | 1 | 1 | 1 | 1 | 6 |
| Simuliidae | 1 | 1 | 1 | 1 | 1 | 1 | 6 |
| Sperchontidae | 1 | 1 | 1 | 1 | 1 | 1 | 6 |
| Taeniopterygidae | 1 | 1 | 1 | 1 | 1 | 1 | 6 |
| Tipulidae | 1 | 1 | 1 | 1 | 1 | 1 | 6 |
| Torrenticolidae | 1 | 1 | 1 | 1 | 1 | 1 | 6 |
| Uenoidae | 1 | 1 | 1 | 1 | 1 | 1 | 6 |
| Aturidae | 1 | 0 | 1 | 1 | 1 | 1 | 5 |
| Blephariceridae | 1 | 1 | 1 | 0 | 1 | 1 | 5 |
| Gammaridae | 1 | 1 | 1 | 1 | 1 | 0 | 5 |
| Peltoperlidae | 1 | 1 | 0 | 1 | 1 | 1 | 5 |
| Stygothrombiidae | 1 | 1 | 0 | 1 | 1 | 1 | 5 |
| Dixidae | 0 | 1 | 0 | 1 | 1 | 1 | 4 |
| Dytiscidae | 0 | 1 | 1 | 1 | 1 | 0 | 4 |
| Hydrozetidae | 0 | 0 | 1 | 1 | 1 | 1 | 4 |
| Hygrobatidae | 0 | 0 | 1 | 1 | 1 | 1 | 4 |
| Lymnaeidae | 0 | 1 | 1 | 1 | 0 | 1 | 4 |
| Pelecorhynchidae | 0 | 1 | 0 | 1 | 1 | 1 | 4 |
| Philopotamidae | 1 | 1 | 0 | 1 | 1 | 0 | 4 |
| Planorbidae | 0 | 1 | 1 | 0 | 1 | 1 | 4 |
| Staphylinidae | 0 | 1 | 1 | 0 | 1 | 1 | 4 |
| Valvatidae | 1 | 1 | 0 | 1 | 1 | 0 | 4 |
| Ametropodidae | 1 | 0 | 1 | 0 | 1 | 0 | 3 |
| Caenidae | 0 | 1 | 1 | 0 | 1 | 0 | 3 |
| Ephemeridae | 1 | 1 | 0 | 0 | 1 | 0 | 3 |
| Feltriidae | 0 | 0 | 1 | 1 | 0 | 1 | 3 |
| Leptohyphidae | 1 | 0 | 1 | 1 | 0 | 0 | 3 |
| Muscidae | 1 | 1 | 1 | 0 | 0 | 0 | 3 |
| Tanyderidae | 1 | 1 | 1 | 0 | 0 | 0 | 3 |
| Amphizoidae | 0 | 0 | 1 | 1 | 0 | 0 | 2 |
| Anisitsiellidae | 1 | 1 | 0 | 0 | 0 | 0 | 2 |
| Crangonyctidae | 0 | 1 | 1 | 0 | 0 | 0 | 2 |
| Curculionidae | 0 | 0 | 0 | 1 | 1 | 0 | 2 |
| Hyalellidae | 0 | 1 | 1 | 0 | 0 | 0 | 2 |
| Hydrophilidae | 0 | 0 | 0 | 1 | 1 | 0 | 2 |
| Isotomidae | 1 | 0 | 0 | 1 | 0 | 0 | 2 |
| Limnesiidae | 1 | 1 | 0 | 0 | 0 | 0 | 2 |
| Mideopsidae | 0 | 0 | 1 | 1 | 0 | 0 | 2 |
| Oreoleptidae | 0 | 0 | 0 | 1 | 1 | 0 | 2 |
| Oxidae | 0 | 1 | 0 | 1 | 0 | 0 | 2 |
| Physidae | 0 | 1 | 0 | 0 | 0 | 1 | 2 |
| Pionidae | 0 | 1 | 1 | 0 | 0 | 0 | 2 |
| Poduridae | 0 | 0 | 1 | 1 | 0 | 0 | 2 |
| Polycentropodidae | 1 | 0 | 0 | 1 | 0 | 0 | 2 |
| Tabanidae | 0 | 1 | 0 | 1 | 0 | 0 | 2 |
| Thaumaleidae | 0 | 1 | 0 | 0 | 1 | 0 | 2 |
| Arrenuridae | 0 | 1 | 0 | 0 | 0 | 0 | 1 |
| Asellidae | 0 | 1 | 0 | 0 | 0 | 0 | 1 |
| Corixidae | 0 | 0 | 0 | 0 | 1 | 0 | 1 |
| Deuterophlebiidae | 0 | 1 | 0 | 0 | 0 | 0 | 1 |
| Dryopidae | 0 | 0 | 0 | 0 | 1 | 0 | 1 |
| Gerridae | 0 | 1 | 0 | 0 | 0 | 0 | 1 |
| Glossiphoniidae | 0 | 1 | 0 | 0 | 0 | 0 | 1 |
| Gomphidae | 0 | 0 | 1 | 0 | 0 | 0 | 1 |
| Halacaridae | 0 | 0 | 1 | 0 | 0 | 0 | 1 |
| Haliplidae | 0 | 1 | 0 | 0 | 0 | 0 | 1 |
| Hydraenidae | 0 | 0 | 0 | 1 | 0 | 0 | 1 |
| Hydrodromidae | 0 | 1 | 0 | 0 | 0 | 0 | 1 |
| Hypogastruridae | 0 | 1 | 0 | 0 | 0 | 0 | 1 |
| Limnocharidae | 0 | 0 | 1 | 0 | 0 | 0 | 1 |
| Margaritiferidae | 0 | 1 | 0 | 0 | 0 | 0 | 1 |
| Piscicolidae | 0 | 1 | 0 | 0 | 0 | 0 | 1 |
| Psychomyiidae | 0 | 0 | 0 | 0 | 0 | 1 | 1 |
| Sialidae | 0 | 1 | 0 | 0 | 0 | 0 | 1 |
| Sminthuridae | 0 | 0 | 0 | 1 | 0 | 0 | 1 |
| Stratiomyidae | 0 | 1 | 0 | 0 | 0 | 0 | 1 |
| Trhypochthoniidae | 0 | 0 | 1 | 0 | 0 | 0 | 1 |
| Unionicolidae | 0 | 1 | 0 | 0 | 0 | 0 | 1 |
| **Total Diversity** | 55 | 77 | 64 | 65 | 62 | 53 |  |

**Supplementary Table 4** – Vancouver Island Taxon Occurrence at Reference Sites

| **Taxon** | **2001_2005** | **2006_2010** | **2011_2015** | **2016_2020** | **nOccur** |
| --- | --- | --- | --- | --- | --- |
| Ameletidae | 1 | 1 | 1 | 1 | 4 |
| Apataniidae | 1 | 1 | 1 | 1 | 4 |
| Aturidae | 1 | 1 | 1 | 1 | 4 |
| Baetidae | 1 | 1 | 1 | 1 | 4 |
| Brachycentridae | 1 | 1 | 1 | 1 | 4 |
| Capniidae | 1 | 1 | 1 | 1 | 4 |
| Ceratopogonidae | 1 | 1 | 1 | 1 | 4 |
| Chironomidae | 1 | 1 | 1 | 1 | 4 |
| Chloroperlidae | 1 | 1 | 1 | 1 | 4 |
| Dixidae | 1 | 1 | 1 | 1 | 4 |
| Elmidae | 1 | 1 | 1 | 1 | 4 |
| Empididae | 1 | 1 | 1 | 1 | 4 |
| Ephemerellidae | 1 | 1 | 1 | 1 | 4 |
| Glossosomatidae | 1 | 1 | 1 | 1 | 4 |
| Heptageniidae | 1 | 1 | 1 | 1 | 4 |
| Hydropsychidae | 1 | 1 | 1 | 1 | 4 |
| Hydroptilidae | 1 | 1 | 1 | 1 | 4 |
| Hydrozetidae | 1 | 1 | 1 | 1 | 4 |
| Hydryphantidae | 1 | 1 | 1 | 1 | 4 |
| Hygrobatidae | 1 | 1 | 1 | 1 | 4 |
| Lebertiidae | 1 | 1 | 1 | 1 | 4 |
| Lepidostomatidae | 1 | 1 | 1 | 1 | 4 |
| Leptophlebiidae | 1 | 1 | 1 | 1 | 4 |
| Leuctridae | 1 | 1 | 1 | 1 | 4 |
| Limnephilidae | 1 | 1 | 1 | 1 | 4 |
| Lumbriculidae | 1 | 1 | 1 | 1 | 4 |
| Naididae | 1 | 1 | 1 | 1 | 4 |
| Nemouridae | 1 | 1 | 1 | 1 | 4 |
| Perlidae | 1 | 1 | 1 | 1 | 4 |
| Perlodidae | 1 | 1 | 1 | 1 | 4 |
| Pisidiidae | 1 | 1 | 1 | 1 | 4 |
| Planorbidae | 1 | 1 | 1 | 1 | 4 |
| Rhyacophilidae | 1 | 1 | 1 | 1 | 4 |
| Simuliidae | 1 | 1 | 1 | 1 | 4 |
| Sperchontidae | 1 | 1 | 1 | 1 | 4 |
| Taeniopterygidae | 1 | 1 | 1 | 1 | 4 |
| Tipulidae | 1 | 1 | 1 | 1 | 4 |
| Torrenticolidae | 1 | 1 | 1 | 1 | 4 |
| Enchytraeidae | 1 | 0 | 1 | 1 | 3 |
| Polycentropodidae | 1 | 0 | 1 | 1 | 3 |
| Dytiscidae | 0 | 1 | 1 | 0 | 2 |
| Isotomidae | 1 | 0 | 0 | 1 | 2 |
| Leptoceridae | 1 | 0 | 1 | 0 | 2 |
| Mideopsidae | 0 | 1 | 1 | 0 | 2 |
| Oxidae | 0 | 0 | 1 | 1 | 2 |
| Philopotamidae | 0 | 1 | 1 | 0 | 2 |
| Planariidae | 1 | 1 | 0 | 0 | 2 |
| Psychodidae | 0 | 1 | 1 | 0 | 2 |
| Tabanidae | 0 | 1 | 0 | 1 | 2 |
| Uenoidae | 0 | 0 | 1 | 1 | 2 |
| Astacidae | 0 | 0 | 0 | 1 | 1 |
| Baetiscidae | 1 | 0 | 0 | 0 | 1 |
| Candonidae | 1 | 0 | 0 | 0 | 1 |
| Curculionidae | 0 | 1 | 0 | 0 | 1 |
| Daphniidae | 0 | 0 | 1 | 0 | 1 |
| Dolichopodidae | 0 | 1 | 0 | 0 | 1 |
| Gammaridae | 0 | 1 | 0 | 0 | 1 |
| Glossiphoniidae | 0 | 0 | 1 | 0 | 1 |
| Helophoridae | 1 | 0 | 0 | 0 | 1 |
| Hydrophilidae | 0 | 1 | 0 | 0 | 1 |
| Hydrovolziidae | 0 | 0 | 1 | 0 | 1 |
| Hypogastruridae | 1 | 0 | 0 | 0 | 1 |
| Leptohyphidae | 0 | 1 | 0 | 0 | 1 |
| Leptoplanidae | 0 | 1 | 0 | 0 | 1 |
| Limnesiidae | 0 | 0 | 0 | 1 | 1 |
| Margaritiferidae | 0 | 0 | 1 | 0 | 1 |
| Oreoleptidae | 0 | 1 | 0 | 0 | 1 |
| Pelecorhynchidae | 0 | 0 | 0 | 1 | 1 |
| Physidae | 0 | 0 | 0 | 1 | 1 |
| Piscicolidae | 0 | 1 | 0 | 0 | 1 |
| Poduridae | 0 | 1 | 0 | 0 | 1 |
| Pyralidae | 1 | 0 | 0 | 0 | 1 |
| Sminthuridae | 1 | 0 | 0 | 0 | 1 |
| Valvatidae | 0 | 1 | 0 | 0 | 1 |
| **Total Diversity** | 49 | 54 | 51 | 48 |  |
